# Bioactive glass S53P4 vs. autologous bone graft for filling defects in patients with chronic osteomyelitis and infected non-unions – a single center experience

**DOI:** 10.5194/jbji-6-73-2021

**Published:** 2021-01-12

**Authors:** Eva Steinhausen, Rolf Lefering, Martin Glombitza, Nikolaus Brinkmann, Carsten Vogel, Bastian Mester, Marcel Dudda

**Affiliations:** 1 Department of Orthopedic and Trauma Surgery, BG Klinikum Duisburg, University of Duisburg-Essen, 47249 Duisburg, Germany; 2 Institute for Research in Operative Medicine (IFOM), University of Witten/Herdecke, Cologne, Germany; 3 Department of Trauma, Hand and Reconstructive Surgery, University Hospital Essen, University of Duisburg-Essen, Essen, Germany

## Abstract

**Introduction**: The goals of osteomyelitis therapy are successful control of infection and reconstruction of the bone. The gold standard for filling defects is the autologous bone graft. Bioactive glass S53P4 is an inorganic bone substitute. We compared the outcome of using bioactive glass (BAG) versus autologous bone graft (AB) in patients with infected non-union. **Methods**: Patients with chronic osteomyelitis and infected non-union who received either bioactive glass or autologous bone grafts between 2013 and 2017 were analyzed retrospectively. The primary endpoint was successful control of infection during follow-up. Secondary endpoints were bone healing, functional outcome, and occurrence of complications. **Results**: Eighty-three patients were analyzed (BAG n=51, AB n=32). Twenty-one patients experienced reinfection (BAG n=15, 29 %; AB n=6, 19 %). Seventy-eight patients achieved full weight bearing (BAG n=47, 92 %; AB n=31, 97 %). Sixty-four patients had complete bone healing at the end of the follow-up period (BAG n=39, 77 %; AB n=25, 78 %). There were no significant differences between the groups with respect to the primary or secondary endpoints. Patients with multidrug-resistant pathogens had a significantly higher rate of incomplete bone healing (p=0.033) and a 3-fold higher risk of complications in both groups. **Conclusions**: Bioactive glass appears to be a suitable bone substitute not only for successful control of infection and defect filling but also for bone healing in cases of infected non-union. In our study, bioactive glass was neither superior nor inferior to autologous bone graft with regard to the primary and secondary endpoints. Further studies with larger numbers of patients are required.

## 1 Introduction

Successful infection control is essential in the treatment of chronic osteomyelitis and infected non-union. Adequate surgical debridement remains key to achieving this goal (Ferguson et al., 2017; Lew and Waldvogel, 2004). Surgical debridement should be accompanied by systemic antibiotic treatment. Problems include low antibiotic concentrations due to inadequate perfusion of the bone and the surrounding soft tissues (van Vugt et al., 2016; Romano et al., 2014; Geurts et al., 2011) as well as the development of antibiotic resistance and the formation of biofilms (van Gestel et al., 2015; Lindfors et al., 2017). High concentrations can be achieved with local treatment, e.g., with gentamicin-containing polymethylmethacrylate (PMMA) beads. Currently, the gold standard in treating chronic osteomyelitis is a two-stage procedure that involves the use of antibiotic-containing PMMA beads in the first procedural stage (Geurts et al., 2011; Lindfors et al., 2017; van Vugt et al., 2019). However, the PMMA beads must be removed in a further operation (Ferguson et al., 2017; Geurts et al., 2011), and, after releasing the antibiotics, they can themselves act as a foreign body, thereby creating a base for bacterial biofilms (Romano et al., 2014; Lindfors et al., 2017; Ferguson et al., 2014; Rahaman et al., 2014).

Bone reconstruction is necessary after successful infection control. Autologous bone graft is the gold standard and has osteogenic, osteoinductive, and osteoconductive properties (Ferguson et al., 2017; Calori et al., 2011; De Long et al., 2007; Egol et al., 2015). However, the volume that can be achieved is limited, and donor site morbidity is considerable (Pape et al., 2010).

Various bone substitutes have been developed in recent years to complement or even replace autologous bone grafting (Egol et al., 2015; Fillingham and Jacobs, 2016; Kurien et al., 2013). An ideal bone substitute should be osteoconductive, osteoinductive, biodegradable, and biocompatible (van Vugt et al., 2016; Calori et al., 2011; Fillingham and Jacobs, 2016). Bone substitutes loaded with antibiotics have been developed for the treatment of bone infections. They usually contain gentamicin (Romano et al., 2014; McNally et al., 2016; Fleiter et al., 2014; Lalidou et al., 2014), or alternatively tobramycin (Ferguson et al., 2014; McKee et al., 2010) or vancomycin (Luo et al., 2016), and make it possible to achieve high local antibiotic concentrations with low systemic levels without incurring the disadvantages of PMMA beads (Ferguson et al., 2017; Lindfors et al., 2017; Ferguson et al., 2014; McNally et al., 2016; Fleiter et al., 2014; McKee et al., 2010; Luo et al., 2016). Clinical evidence in this area is still limited, however (Ferguson et al., 2017; Geurts et al., 2011).

Bioactive glass S53P4 is an inorganic bone substitute with antibacterial, osteoconductive, osteostimulative, and angiogenic properties (Cunha et al., 2018; Coraca-Huber et al., 2014). The results of existing clinical studies of bioactive glass are promising (van Gestel et al., 2015; Lindfors et al., 2017; Ferrando et al., 2017; Lindfors et al., 2010; Auregan and Begue, 2015).

Here, we compare the outcome of bioactive glass S53P4 versus autologous bone grafts for filling defects in patients with chronic osteomyelitis and infected non-union.

## 2 Methods

### 2.1 Bioactive glass S53P4 (BAG)

Bioactive glass (BAG) (Bioglass S53P4; BonAlive^®^ Biomaterials Ltd., Turku, Finland) is an inorganic bone substitute containing SiO${}_{2}$, Na${}_{2}$O, CaO, and P${}_{2}$O${}_{5}$. Following implantation, sodium and basic ions are released, causing a rapid increase in pH and osmotic pressure. A silicon dioxide-rich layer is formed on the surface. Calcium and phosphate groups migrate to the surface to form a hydroxyl-apatite scaffold. Osteoblasts migrate into this scaffold, proliferate, differentiate, and stimulate osteogenesis. These surface reactions not only stimulate the formation of new bone, but also have an antibacterial effect (Lindfors et al., 2017; Coraca-Huber et al., 2014; Lindfors et al., 2010). In addition, in vitro studies have shown an angiogenic effect with increased secretion of the vascular endothelial growth factor (VEGF) (Detsch et al., 2014).

### 2.2 Patient collective

We conducted a retrospective analysis of patients treated in our institution for chronic osteomyelitis and infected non-union who underwent filling of a bone defect with either autologous bone graft or BAG between July 2013 and January 2017.

**Figure 1:**
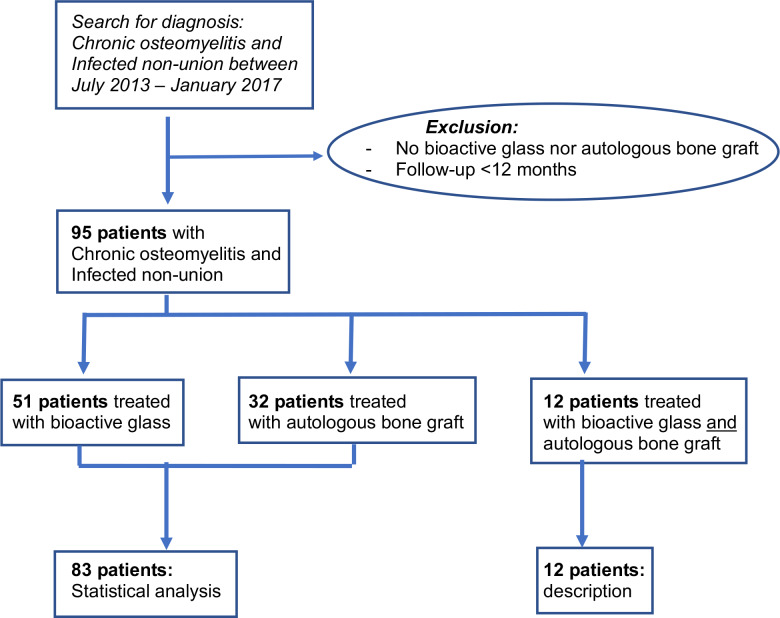
Flowchart of the patient recruitment process.

Inclusion criteria were age > 18 years, chronic osteomyelitis and infected non-union (positive bacteriology and/or positive histology), filling of the bone defect with either autologous bone graft or BAG, and minimum follow-up of 12 months. Exclusion criteria were aseptic non-unions and lack of follow-up (Fig. 1).

Descriptive epidemiologic data and details of the pre-, peri-, and post-operative courses were compiled from the digital patient files and processed anonymously. Follow-up was analyzed up to a January 2018 cut-off.

The primary endpoint was successful control of infection during follow-up (no clinical, laboratory or radiological signs of infection, closed soft tissues). Secondary endpoints were bone healing and functional outcome focusing on mobilization with full weight bearing. Any complications were recorded.

Bone healing was quantified by evaluating X-ray or CT imaging. Healing was defined as the presence of complete cortical bridging in three out of four cortices or with no evidence of the fracture line.

Multi-resistant pathogens were defined as pathogens resistant to most antibiotics or antibiotic groups (*methicillin-resistant Staphylococcus aureus, multi-resistant coagulase-negative Staphylococci, multidrug-resistant Gram-negative bacteria and extended spectrum ß-lactamase bacteria*).

### 2.3 Surgery

All patients were treated exclusively by the specialized team of the septic surgery department. Decisions regarding treatment method were taken individually for each patient based on surgeon preference.

In all cases, radical debridement was carried out prior to filling the defect. The surgeons took care to completely fill the defect and to press the implanted BAG into the surrounding bone. New samples were taken intraoperatively from all patients.

All patients received antibiotic prophylaxis perioperatively for previously detected pathogens. If an unknown pathogen was present, the patients received cefazoline as the standard antibiotic used in our institution. Patients with an uncomplicated course and sterile samples did not receive postoperative antibiotic treatment. Patients with a prolonged course with multiple previous revisions or with persistent detection of pathogens received postoperative antibiotic treatment. Antibiotic treatment was chosen according to the pathogen and the antibiotic resistance pattern. Antibiotics were given intravenously for 3 weeks and orally for an additional 3 weeks.

### 2.4 Statistics

Treatment groups were compared using Fisher's exact test for binary data and the Mann–Whitney U test for continuous data. A p value <0.05 was considered statistically significant. Given the sample size of about 80 cases, the power of this study is limited, especially for even counts. Significant differences for a theoretical prevalence rate of 20 % could be expected only if the prevalence was < 5 % or > 40 % in the other group. For continuous measures, the detectable difference was about half a standard deviation. Statistical analysis was performed using SPSS (Version 24, IBM Inc., Armonk, NY, USA). Multivariate analyses were conducted for complications, recurrence of infection, and osseous fusion.

## 3 Results

Fifty-one patients received bioactive glass S53P4 (BAG), 32 patients autologous bone graft (AB), and 12 patients a combination of bioactive glass and autologous bone graft. The outcomes for these 12 patients are described separately at the end of this section but are not analyzed statistically. Therefore, the following results represent a total of 83 patients (Fig. 1).

### 3.1 Preoperative

The autologous bone graft and the bioactive glass group were comparable with respect to sex, localization and kind of fracture, and the number of previous operations and flaps (Table 1). However, the patients of the BAG group were significantly older and had undergone an unsuccessful prior attempt at defect filling significantly more often than those in the autologous bone graft group. *Staphylococcus aureus* and coagulase-negative Staphylococci were the most common pathogens involved in both groups. Gram-positive pathogens were found significantly more often in the bioactive glass group. Gram-negative pathogens were detected in both groups without significant difference. Multiple pathogens and multidrug-resistant pathogens also did not differ significantly between the groups (Tables 1, 2).

**Table 1:** Epidemiologic data and preoperative findings.

	Total	Autologous	Bioactive	p value
		bone graft	glass S53P4	
Total number of patients	83	32	51	
Sex (male, %)	61 (74 %)	25 (78 %)	36 (71 %)	0.61
Mean age (y), SD (range)	52.4 ± 12.7	45.9 ± 13.1	56.5 ± 10.7	0.001
Lower extremity (n, %)	78 (94 %)	29 (91 %)	49 (96 %)	0.31
Open fracture (n, %)	28 (34 %)	13 (41 %)	15 (29 %)	0.34
No. of previous operations	21/38/24	9/12/11	12/26/13	0.79
(< 5/5–10/> 10)	(25%/46%/29%)	(28%/38%/34%)	(24%/51%/25%)	
Flaps prior to arthrodesis (n, %)	30 (36 %)	8 (25 %)	22 (43 %)	0.11
Previous defect filling	18 (22 %)	2 (6 %)	16 (31 %)	0.007
Multiple pathogens (n, %)	34 (41 %)	13 (41 %)	21 (41 %)	0.54
Multidrug-resistant pathogens (n, %)	27 (33 %)	14 (44 %)	13 (26 %)	0.097
Gram-positive pathogen (n, %)	62 (75 %)	18 (56 %)	44 (86 %)	0.004
*Staphylococcus aureus* (n, %)	30 (36 %)	8 (25 %)	22 (43 %)	0.107

### 3.2 Perioperative

In the BAG group, an average of 11 cm${}^{3}$ bioactive glass was implanted (minimum 5 cm${}^{3}$; maximum 30 cm${}^{3}$). Intraoperative samples showed persistent bacteria in 17 patients of the BAG group and in 11 patients of the AB group, indicating no significant difference between the groups. However, patients of the autologous bone graft group had undergone a change in pathogens significantly more often (BAG 3.9 %; AB 28.1 %; p=0.001). Forty patients of the BAG group and 29 patients of the AB group received postoperative antibiotic treatment owing to a prolonged course or persistent detection of pathogens. No significant differences were found between the two groups regarding postoperative antibiotic treatment (Table 3).

**Table 2:** Causative pathogens identified preoperatively and their proportions.

Pathogen	Bioactive glass	Autologous bone graft
Total number of patients with known pathogens	n=45	n=26
Total number of positive microbiology findings${}^{a}$	n= 79 (100 %)${}^{b}$	n= 39 (100 %)${}^{b}$
Staph. aureus	n= 22 (28 %)	n= 8 (21 %)
COST	n= 22 (28 %)	n= 11 (28 %)
MRSA	n= 2 (2 %)	n= 3 (7 %)
Streptococcus	n= 6 (8 %)	n= 0 (0 %)
Enterococcus	n= 5 (6 %)	n= 1 (3 %)
Enterobacter	n= 3 (4 %)	n= 2 (5 %)
Proteus	n= 1 (1 %)	n= 1 (3 %)
Serratia	n= 3 (4 %)	n= 1 (3 %)
Pseudomonas	n= 6 (8 %)	n= 0 (0 %)
E. coli	n= 3 (4 %)	n= 5 (12 %)
Others	n= 6 (8 %)	n= 7 (18 %)

COST: coagulase-negative Staphylococci; MRSA: methicillin-resistant *Staph. aureus*; *E. coli: Escherichia coli*. ${}^{a}$ Multiple nominations per patient possible. ${}^{b}$ Percentages are given in relation to all positive microbiology findings, not in relation to the number of patients; therefore, differences are possible compared to Table 1 (percentages in relation to the number of analyzed patients).

### 3.3 Postoperative

Patients of the autologous bone graft group had significantly longer follow-up than patients of the bioactive glass group (p<0.001).

Overall, major and minor complications were found in 22 patients of the BAG group and in 20 patients of the AB group, indicating no significant difference between the groups. Recurrence of infection occurred in 15 patients of the BAG group and in 6 patients of the AB group. Statistical analysis also failed to show a significant difference between the groups with regard to recurrence of infection. Two patients (6.3 %) suffered complications requiring revision surgery after removal of autologous bone from the iliac crest. No complications associated with bioactive glass were observed.

Forty-seven patients of the BAG group and 31 patients of the AB group achieved full weight bearing during follow-up, indicating no significant difference in this respect. However, patients of the BAG group achieved full weight bearing significantly more rapidly (BAG 5.9 ± 4.1 months, median 6 months; AB 10.7 ± 8.6 months, median 10 months; p=0.018).

The results for bone healing were similar in both groups, with bone healing being seen in 39 patients of the BAG group and in 25 patients of the AB group by the end of follow-up. Again, patients in the BAG group accomplished bone healing more rapidly (BAG 9.5 ± 7.0 months, AB 10.8 ± 9.0 months). Eleven patients of the AB group underwent another procedure to fill the defect, significantly more frequently than in the BAG group (BAG n=8; p=0.049). In addition, patients of the autologous bone graft group underwent reoperations significantly more frequently (BAG n=24; AB n=24; p=0.014). The rate of below-knee amputation due to recurrent infection during follow-up was comparable between the two groups (BAG n=3; AB n=2; p=1.00) (Table 3).

**Table 3:** Peri- and post-operative findings.

	Total	Autologous	Bioactive	p value
		bone graft	glass S53P4	
Number of patients	83	32	51	
Perioperative findings				
Persistent bacteria intraoperative (n, %)	28 (34 %)	11 (34 %)	17 (33 %)	1.00
Change in pathogens (n, %)	11 (13 %)	9 (28 %)	2 (4 %)	0.001
Postoperative antibiotic treatment (n, %)	69 (83 %)	29 (91 %)	40 (78 %)	0.23
Postoperative findings				
Follow-up (mean in months; median)	24.7 ± 11.8; 21	31.3 ± 12.7; 30	20.5 ± 9.1; 18	< 0.001
Recurrence of infection (n, %)	21 (25 %)	6 (19 %)	15 (29 %)	0.31
Major and minor complications (n, %)	42 (51 %)	20 (63 %)	22 (43 %)	0.12
Bone fusion (n, %)	64 (77 %)	25 (78 %)	39 (77 %)	1.00
Full weight bearing (n, %)	78 (94 %)	31 (97 %)	47 (92 %)	0.65
Further operations (n, %)	48 (58 %)	24 (75 %)	24 (47 %)	0.014
Additional defect filling (n, %)	19 (23 %)	11 (34 %)	8 (16 %)	0.049
Amputation (n, %)	5 (6 %)	2 (6 %)	3 (6 %)	1.00

### 3.4 Multivariate analyses

Detection of multi-resistant pathogens was associated with a significantly higher rate of incomplete osseous fusion (p=0.033) and was also associated with a 3-fold increase in the risk of complications (odds ratio 3.091, 95 %-confidence interval 1.128–8.470). Male patients had a significantly greater risk of recurrent infection. Finally, there was a 3.5-fold increased risk of recurrent infection after local or free tissue transfer (odds ratio 3.508, 95 %-confidence interval 1.155–10.660).

### 3.5 Combination of autologous bone graft and bioactive glass

Twelve patients had a defect filled with a combination of BAG and autologous bone graft. Intraoperative samples revealed persistent bacteria in two patients (16.7 %). Full weight bearing was reached after 4.8 months, on average. Nine patients (75 %) achieved complete bone healing during follow-up after an average of 7.6 months. Four patients (33.3 %) developed complications. Only one patient (8.3 %) had recurrent infection during follow-up.

## 4 Discussion

The goals of osteomyelitis therapy are successful control of infection and the reconstruction of the bone. Bioactive glass S53P4 is an inorganic bone substitute with antibacterial, osteoconductive, and osteostimulative properties (Coraca-Huber et al., 2014; Heikkila et al., 1995).

Our comparison of the outcomes of patients with chronic osteomyelitis and infected non-union who had a defect filled with either autologous bone graft or BAG revealed no significant differences between the groups with respect to recurrence of infection, bone healing, full weight bearing, or complications in general. Some studies have investigated the use of BAG in patients with chronic osteomyelitis. However, the number of cases in these studies was usually small, and most had no control group (Romano et al., 2014; Lindfors et al., 2017, 2010; Auregan and Begue, 2015; McAndrew et al., 2013; Drago et al., 2013; Malat et al., 2018; Oosthuysen et al., 2020). Existing comparative studies compare BAG either with other bone substitutes (Table 4) or with PMMA beads (the gold standard for a two-stage procedure) and with the objective of investigating successful control of infection and dead space management of bone cavities, but not bone healing. To our knowledge, ours is the first study to compare BAG with autologous bone graft in patients with an infected non-union that includes fracture healing as a secondary endpoint.

**Table 4:** Clinical studies investigating the use of bioactive glass S53P4 in patients with chronic osteomyelitis; reviews and animal and in vitro studies are not included.

Author	Year	No. of	Bone	Persistent or	Comment
		patients	substitute	reinfection	
Lindfors	2017	116	BAG	12 (10.3 %)	No control group
Lindfors	2010	11	BAG	1 (9.1 %)	No control group
Drago	2013	27	BAG	3 (11.1 %)	No control group
McAndrew	2013	3	BAG	0 (0 %)	No control group
Romano	2014	76	BAG (n= 27) vs. antibiotic-loaded HA and calcium sulfate (n= 27) vs. antibiotic-loaded demineralized bone matrix and tricalcium phosphate (n= 22)	2 (7.4 %) 3 (11.1 %) 3 (13.6 %)	No significant differences
Ferrando	2017	25	BAG (n= 12) vs. calcium sulfate antibioticbeads (n= 13)	1 (8.3 %) 1 (7.7 %)	No significant differences
Malat	2018	50	BAG	7 (14 %)	No control group
Oosthuysen	2019	24	BAG	2 (8 %)	No control group

HA: hydroxyapatite; BAG: bioactive glass.

### 4.1 Control of infection

The reinfection rate of patients with chronic osteomyelitis is reportedly as high as 21.2 % (Walenkamp et al., 1998), and the overall reinfection rate after implantation of BAG is 0 %–14 % according to the current literature (Romano et al., 2014; van Gestel et al., 2015; Lindfors et al., 2017, 2010; McAndrew et al., 2013; Drago et al., 2013; Malat et al., 2018; Tanwar and Ferreira, 2020). We saw higher reinfections rates, especially in the BAG group. However, the number of patients treated with BAG in published studies is usually small. Moreover, the definition of reinfection or persistence of infection used by other authors is less strict than ours. Romano et al. (2014) defined a “fair” outcome as a wound with prolonged drainage or serum leakage of up to 6 weeks and a “poor” outcome as no wound healing for more than 6 weeks or one requiring surgical intervention. Lindfors et al. (2017) defined the outcomes of their patients in a similar way. These findings would be interpreted as persistent infection in our assessment. These differences in definition may explain the increased infection rate in our BAG group. On the other hand, the infection rate of our autologous bone graft group correlates with that reported in other studies. *Staphylococcus aureus *and *coagulase-negative Staphylococci* were the most common pathogens involved in both groups. The presence of multi-resistant pathogens was associated with an increased risk of complications regardless of treatment. These results agree with those in other publications. An additional risk factor for recurrent infection in both groups was a previous local or free tissue transfer, as also reported by Lindfors et al. (2017).

All factors considered, we could not establish an advantage of BAG compared to AB with regard to infection control. There were no significant differences between our two groups in other respects; that is, we found no clear disadvantage for BAG.

### 4.2 Bone healing and mobilization with full weight bearing

**Figure 2:**
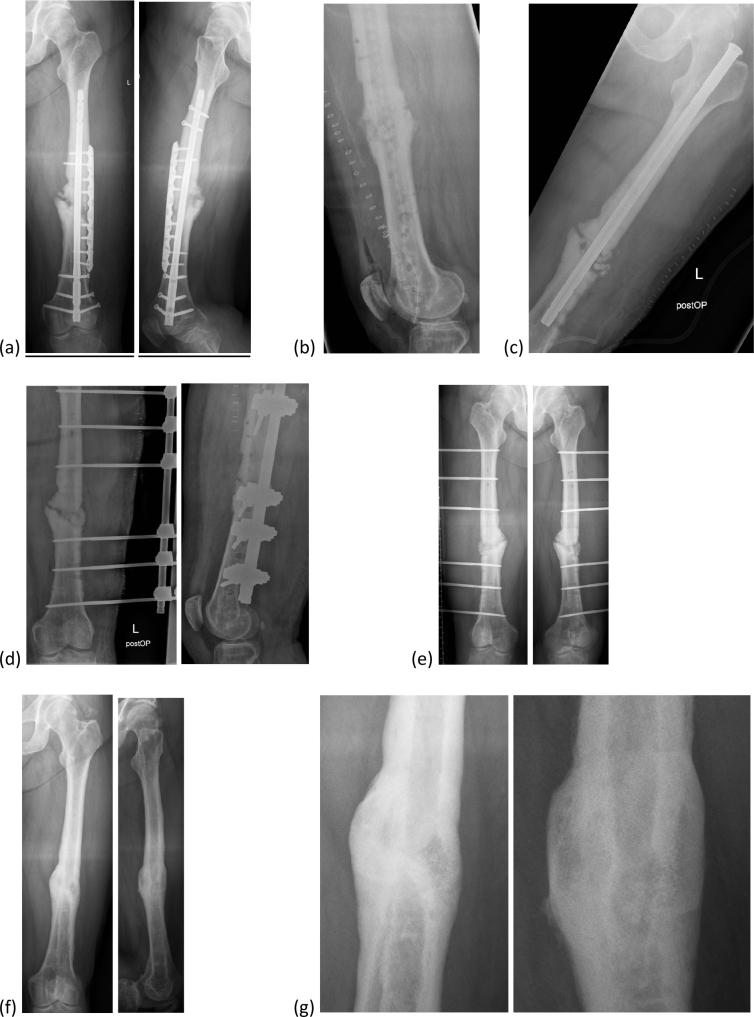
Bioactive glass in a patient with an infected non-union of the femur. **(a)** Infected non-union of the femur with loosened retrograde intramedullary nail and plate osteosynthesis. **(b)** After debridement with removal of the internal osteosynthesis, reaming of the intramedullary canal and insertion of a PMMA chain. **(c)** Temporary stabilization with an antegrade intramedullary nail. **(d)** Defect filling with bioactive glass after control of infection; definitive stabilization with external fixator. **(e)** Six months after defect filling. **(f)** Final result with bony fusion after 2 years. **(g)** Image enlargement of **(f)**.

Our rate of complete osseous fusion is 77.1 % without significant difference between the groups at latest follow-up. Published studies do not report comparable rates (Romano et al., 2014; van Gestel et al., 2015; Lindfors et al., 2017; Ferrando et al., 2017; Lindfors et al., 2010; McAndrew et al., 2013; Drago et al., 2013). This is unsurprising because these studies investigated the use of BAG only as a bone void filler in chronic osteomyelitis. However, they describe progressive incorporation of BAG, and radiographs therein show partial incorporation at latest follow-up, although the biomaterial remained visible (Romano et al., 2014). In addition, they describe the formation of a thickened cortex (van Gestel et al., 2015). BAG was not fully incorporated in our patients, either, and we observed the phenomenon of a thickened cortex (Fig. 2).

Ninety-four percent of our patients reached full weight bearing. Thus, we assume that, although osseous fusion remained incomplete on some radiographs, bone consolidation was adequate to ensure stability. Basically, these patients regained a working extremity.

### 4.3 Complications

We observed high complication rates in both groups. The actual rate of reinfection was higher in the BAG group, but patients of the AB group needed significantly more reoperations and significantly more additional defect fillings. Our rate of complications from harvesting autologous bone grafts from the iliac crest is comparable with that reported in other studies (Dimitriou et al., 2011). However, only complications requiring revision surgery are mentioned in our study. Pain after removal of autologous bone graft from the iliac crest and the resulting delay in mobilization are not recorded, although nearly all patients suffer these complications. Last but not least, harvesting an autologous bone graft from the anterior or posterior iliac crest requires prolonged surgery. These drawbacks do not apply to the use of bone substitutes. In our study, bioactive glass was not associated with adverse effects. This matches the experience in the other published studies. So far, there has been no report of BAG-associated complications or formation of bacterial resistance (Romano et al., 2014; van Gestel et al., 2015; Drago et al., 2013). The tolerability of BAG is even described as superior when compared to other bone substitutes (Ferguson et al., 2014; McNally et al., 2016). Van Gestel et al. (2015) referred to bioactive glass S53P4 as a potential new gold standard.

### 4.4 One-stage versus two-stage procedure

Lindfors et al. (2017) analyzed the largest series of patients (n=116) with chronic osteomyelitis to date, but without a control group. BAG was used in a one-stage procedure in most of the cases, with excellent results. The authors highlight the possibility of one-stage use due to the antibacterial effect as the key advantage of BAG. Other authors agree and underscore its angiogenetic and antibacterial effects (van Gestel et al., 2015; McAndrew et al., 2013; Drago et al., 2013), although radical debridement remains indispensable (Lew and Waldvogel, 2004; Lindfors et al., 2017; Simpson et al., 2001).

Geurts et al. (2011) compared various antibiotic-loaded bone graft substitutes in active or suspected infection with PMMA beads. The authors argue that PMMA beads remain the gold standard even if they require a two-stage procedure. Hence, new biomaterials show great potential, but the level of available evidence is still limited. A recently published systematic review has identified a wide range of successful single-stage procedures for the treatment of chronic osteomyelitis including the use of BAG (Pincher et al., 2019). Finally, a recent study has demonstrated the cost-effectiveness of one-stage treatment of chronic osteomyelitis with the bioactive glass S53P4 (Geurts et al., 2019). However, our procedure is different for both BAG and autologous bone graft. We repeated surgical debridement until the intraoperative samples were sterile, in large part because most of our patients had a protracted and therapy-refractory course.

### 4.5 Antibacterial effect

In contrast to autologous bone graft, BAG has antibacterial and angiogenetic effects. These effects are especially advantageous in often poorly vascularized bone. Previous studies show that bacterial biofilms can cause the infecting organism to be resistant to systemic antibiotic concentrations of up to 1000 times greater than normal therapeutic levels (McKee et al., 2010). Such concentrations cannot be achieved with systemic application.

Recently, Ferrando et al. (2017) compared bioactive glass S53P4 (n=12) with calcium sulfate antibiotic beads (n=13). They did not find significant differences with regard to complication rates and recurrence of infection, but the number of patients was small. The authors conclude that BAG without adjunctive use of local antibiotics is as effective as calcium sulfate antibiotic beads.

Van Vugt et al. (2016) evaluated the use of various bone graft substitutes (Osteoset T, BAG, PerOssal, Herafill-G) in a systematic review (n=15 studies). In general, they criticize weak study design, small number of patients, low levels of evidence (LoE 2b-3b), and a high risk of bias. Interestingly, the reinfection rate (primary outcome) was higher in high-quality studies. No significant differences were found between the different bone graft substitutes. The authors conclude that the results are inconclusive.

To our knowledge, no study to date compares or proves the superiority of BAG over autologous bone graft with respect to its antibacterial effect.

### 4.6 BAG in combination with autologous bone graft

**Figure 3:**
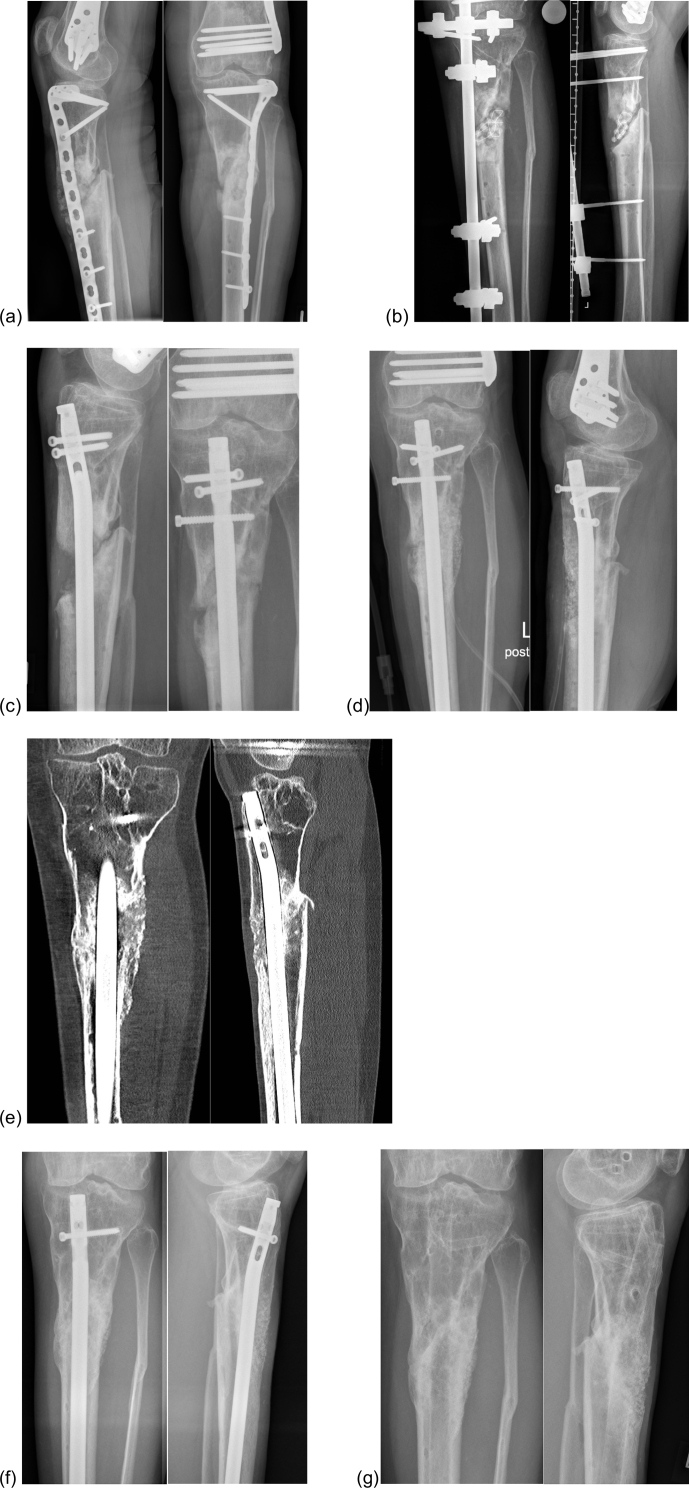
Bioactive glass in combination with autologous bone graft in a patient with an infected non-union of the proximal tibia. **(a)** Infected non-union of the proximal tibia and plate osteosynthesis. **(b)** After debridement with removal of the internal osteosynthesis, resection of the infected bone and implantation of a PMMA chain. **(c)** Re-osteosynthesis with an intramedullary nail after control of infection. **(d)** Defect filling with a combination of bioactive glass S53P4 and autologous bone graft. **(e)** Twelve months after defect filling. **(f)** Two years after defect filling: bony fusion. **(g)** Final result. Abbreviations: AB: autologous bone graft; BAG: bioactive glass; PMMA: polymethylmethacrylate; VEGF: vascular endothelial growth factor.

We combined bioactive glass S53P4 with autologous bone graft in treating large bone defects (n=12) (Fig. 3). The current literature contains no comparable in vivo studies. An in vitro study has analyzed and verified the antibacterial activity of BAG as a bone graft extender in combination with AB (Bortolin et al., 2018). However, to date there is no evidence to indicate which mixing ratio of bioactive glass and autologous bone graft is optimal. The optimal technique – in layers versus mixed – also remains to be determined. Yet our results in a small number of cases are excellent with respect to our primary and secondary endpoints. Therefore, we prefer the combination of BAG and AB for the treatment of large bone defects. However, further evidence in favor of this approach is needed.

### 4.7 Limitations

Our study has some limitations. First, it is a retrospective observational study – although with a control group. The groups are of different sizes, and patients in the BAG group are significantly older. The level of evidence is weak. The maximum follow-up differs between the groups, with significantly longer follow-up for the AB group. However, most complications occurred in the 12 months after surgery, so the difference in length of follow-up is not expected to have a significant effect on the observed rate of complications.

## 5 Conclusions

To our knowledge, this is the first study to compare the use of BAG and AB in patients with chronic osteomyelitis and infected non-union. The results are promising. BAG seems to be an appropriate bone substitute, not only for filling bone defects in patients with chronic osteomyelitis, but also for achieving fracture healing in cases of infected non-union. In our study, BAG was neither superior nor inferior to autologous bone graft with regard to our primary and secondary endpoints. Prospective randomized studies – as recommended by many authors – would be desirable but are not truly feasible. An analysis with matched pairs may be an alternative.

## Ethical statement

This study was favorably evaluated by the Ethics Committee of Witten/Herdecke University (no. 248/2017).

The study has been performed in accordance with the principles of the Declaration of Helsinki.

We obtained informed consent from the analyzed patients.

## Data Availability

The datasets analyzed during the current study are available from the corresponding author on reasonable request.
